# An Analytical Model for CMUTs with Square Multilayer Membranes Using the Ritz Method

**DOI:** 10.3390/mi7040055

**Published:** 2016-03-29

**Authors:** Wen Zhang, Hui Zhang, Shijiu Jin, Zhoumo Zeng

**Affiliations:** State Key Laboratory of Precision Measurement Technology and Instrument, Tianjin University, Tianjin 300072, China; baswen@tju.edu.cn (W.Z.); shjjin@tju.edu.cn (S.J.); zhmzeng@tju.edu.cn (Z.Z.)

**Keywords:** capacitive micromachined ultrasonic transducer (CMUT), multilayer membrane, static deflection, frequency response, residual stress compensation

## Abstract

Capacitive micromachined ultrasonic transducer (CMUT) multilayer membrane plays an important role in the performance metrics including the transmitting efficiency and the receiving sensitivity. However, there are few studies of the multilayer membranes. Some analytical models simplify the multilayer membrane as monolayer, which results in inaccuracies. This paper presents a new analytical model for CMUTs with multilayer membranes, which can rapidly and accurately predict static deflection and response frequency of the multilayer membrane under external pressures. The derivation is based on the Ritz method and Hamilton’s principle. The mathematical relationships between the external pressure, static deflection, and response frequency are obtained. Relevant residual stress compensation method is derived. The model has been verified for three-layer and double-layer CMUT membranes by comparing its results with finite element method (FEM) simulations, experimental data, and other monolayer models that treat CMUTs as monolayer plates/membranes. For three-layer CMUT membranes, the relative errors are ranging from 0.71%–3.51% for the static deflection profiles, and 0.35%–4.96% for the response frequencies, respectively. For the double-layer CMUT membrane, the relative error with residual stress compensation is 4.14% for the central deflection, and −1.17% for the response frequencies, respectively. This proposed analytical model can serve as a reliable reference and an accurate tool for CMUT design and optimization.

## 1. Introduction

Capacitive Micromachined Ultrasonic Transducers (CMUTs) were introduced to the ultrasound community about three decades ago [1,2]. Compared to traditional piezoelectric transducers, CMUTs exhibit some attractive features such as lower mechanical impedance [3], broader bandwidth [4], lower sensitivity to temperature [5], stable device properties, ease of fabrication, and compatibility with Micro-Electro-Mechanical Systems (MEMS) devices [6]. Thus, they have become an excellent choice suitable for a wide range of applications including medical diagnostic imaging [7,8], nondestructive testing [9], and automotive collision avoidance applications such as parking assistance or blind-spot monitoring [10]. In order to facilitate the design and optimization of CMUTs, numerous investigations have been directed towards understanding, predicting and controlling their static and dynamic characterizations [11,12].

**Figure 1:**
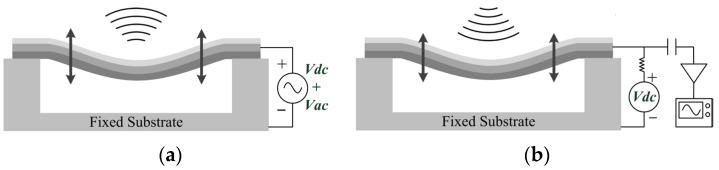
Working principles of a multilayer membrane capacitive micromachined ultrasonic transducer (CMUT) (**a**) as a transmitter and (**b**) as a receiver.

A typical CMUT is built with a square, rectangular, circular, or hexagonal membrane separated from a fixed substrate by a small airgap [13,14]. The working principles are illustrated in Figure 1. The vibrating membrane can generate or detect ultrasonic waves as a transmitter or a receiver. During transmit-mode operation, a DC voltage is applied between the two electrodes. The membrane is attracted to the fixed substrate by an electrostatic force. When an AC voltage is superimposed over the DC voltage, the membrane will move in response to the applied signal, generating an ultrasonic wave that is launched into the ambient environment. During the receive-mode operation, the CMUT membrane is subjected to an ultrasonic wave. The membrane deformation will occur and produce a capacitance variation. A microelectronic circuit is used to detect the capacitance variation and process this into a useful signal.

Because the CMUT’s performances depend on the change of the deflection before and after that the membrane is biased, accurate calculation of the deflection is crucial [15]. Besides, the response frequency, as an important dynamic characteristic, determines the specific application and operational bandwidth of the device [16]. Therefore, we have chosen the static deflection and the response frequency as the research focuses of this paper.

Since the exact shape of a deformed clamped membrane is not known, generalized plate theories have been applied by many authors to obtain a functional form of the membrane deformation curve. The deflection calculation must satisfy the boundary conditions, membrane geometry, and the specific loading condition. As is known that the boundary condition depends on specific membrane geometry (e.g., square, rectangular, circular, or hexagonal), the load deflection model and deflection functions are unique for each of the cases and each demands separate treatment. For clamped square and rectangular membranes, no simple exact deflection solution exists and approximate methods must be used. In this paper, only the square membrane has been considered.

The first CMUTs were built using a sacrificial release process that has become the standard CMUT fabrication method. Numerous variations of the sacrificial release process have been published, all based on the same basic principle [2,5]. The wafer-bonding method is another widely-used CMUT fabrication technique nowadays, based on a different approach to cavity formation that uses a combination of bulk and surface micromachining techniques [2,13]. Commonly, the membrane is fabricated directly using a thin conduction film (e.g., aluminum or polysilicon) or a composite of a non-conduction film (e.g., silicon nitride) with a conduction coating on top (e.g., aluminum or gold). To avoid electrical breakdown after the pull-in phenomenon, an insulation layer is added, either under the membrane or on top of the fixed substrate. Additionally, a passivation layer is fabricated on top of the membrane to protect the CMUT from environmental contamination [13]. The fabrication process described above determines the multilayer structure of the CMUT membrane.

Researchers have described a variety of approaches to determine the deflection curve of single-layer clamped square membranes used in MEMS devices. Timoshenko and Woinowsky-Krieger presented an approximate mathematical expression to determine the deflection curve for thin plate in the small deflection regime using a trigonometric series [17]. However, the model is computationally expensive because it requires extensive numerical calculations to determine a set of coefficients. Bao provided the mechanical analysis of the square membrane, from which the deflection profile and the vibration frequency could be obtained [6]. Ben Moussa used the Galerkin method with a polynomial basis function to determine the deformation curve [18]. This model is limited in accuracy and exhibits slow convergence. Kerrour and Hobar improved the accuracy and convergence of the approach in [19] by replacing the polynomial basis function with a trigonometric basis function [19]. However, the model has not been verified against finite element method (FEM) or experimental results. Elgamel proposed a deflection shape function using a single-term Fourier approximation of the exact bending shape that incorporates a double cosine square term [20]. Because of its simpler form, this function is widely used for load-deflection analysis of clamped square membranes subject to large deflection. However, its accuracy is compromised by the truncation of higher order terms in the Fourier series. Rahman *et al.* developed a deflection shape function by modifying a polynomial-based function presents in [18] to include two empirically determined parameters to improve the accuracy, and the analytical results were compared with FEM and experiments for the single-layer membrane [15].

However, there is a lack of research focusing on multilayer characterization. Analytically, many current derivations treat the multilayer membrane as a monolayer plate/membrane, and are dependent mainly on the properties of the device layer [2,16,21,22]. In fact, when the multilayer membrane is under the action of external forces, each layer suffers deformation and internal forces developed between each layer. In such cases, material coupling will occur due to transverse bending and in-plane stretching. Obviously, such simplification will lead to inaccuracies and errors in the strain-displacement equations of the membrane. FEM has also been widely used to understand transducer characteristics and to optimize transducer response, since FEM results are highly accurate and reliable. However, in most applications, the thickness of the CMUT’s membrane is small in comparison with its lateral dimensions. Since the FEM mesh size is limited to a minimum size, the finite element simulation may have a huge mesh generation with a poor element quality. Also, the simulation could take a long time to make a single change in one parameter. Since most CMUTs have regular shapes with limited boundary conditions, finite element simulations are not efficient for design optimization. In contrast, analytical models can provide better insight into the multilayer characteristics. Therefore, it is necessary to establish an accurate analytical model for the multilayer characterizations of CMUTs.

In this paper, we presented a new analytical model which focuses on the CMUT’s multilayer characterization. The proposed model offers (1) a new, readily usable, simple, and accurate deflection shape function for uniformly loaded clamped square multilayer membrane used in typical CMUT design; (2) a response frequency calculation model derived under the external pressure variation; and (3) a residual stress compensation method based on energy functional using the Ritz method. The rest of the paper has been organized in the following order: Section 2 derives the proposed energy functional model and the compensation method based on the Ritz method and Hamilton’s principle. The relationship between the external pressure, the static deflection, and the response frequency is obtained Section 3 provides the experimental and FEM validation of the model for both the three-layer and double-layer CMUT membranes. Analytical derivations with and without the residual stress compensation have also been compared for the double-layer CMUT membrane. Section 4 concludes the paper and assesses the validity of the proposed model.

## 2. Analytical Modeling

**Figure 2:**
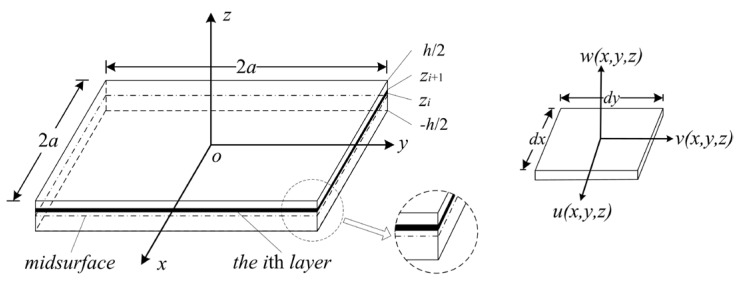
A schematic drawing of a multilayer CMUT membrane.

Figure 2 shows a schematic drawing of a multilayer membrane used in CMUTs. The vibrating section is a clamped square *N*-layer membrane that is excited by an electrostatic force. The origin *O* of the Cartesian coordinate system is in the center, and the *xoy* surface is in the middle layer (the “midsurface”) of the multilayer membrane. 2*a* and *h* are the width and the total thickness. The top and bottom surfaces of the membrane are located at *h*/2 and −*h*/2 in the *z* direction. The *i*th layer is located between *z_i_* and *z_i_*_+1_. Here, *E_i_*, *ρ_i_*, and *μ_i_* are Young’s Modulus, the density, and Poisson’s ratio of the *i*th layer, respectively.

### 2.1. Static Deflection Analysis

Under Kirchhoff’s hypothesis, the inplane displacements *u*, *v* and the transverse deflection *w* of the membrane in the *x*, *y*, and *z* directions are:

(1) $$\left\{ \begin{array}{l} u\left(x,y,z\right)=u_{0}\left(x,y\right)+\lambda_{x}z\\ v\left(x,y,z\right)=v_{0}\left(x,y\right)+\lambda_{y}z\\ w\left(x,y,z\right)=w_{0}\left(x,y\right) \end{array}\right.$$

where $u_{0}\left(x,y\right)$, $v_{0}\left(x,y\right)$, and $w_{0}\left(x,y\right)$ are the displacement components in the midsurface in the *x*, *y*, and *z* directions. $\lambda_{x}$ and $\lambda_{y}$ are the rotations of the midsurface about the *x/y* axis given by:

(2) $$\left\{ \begin{array}{l} \lambda_{x}=-\frac{\partial w_{0}}{\partial x}\\ \lambda_{y}=-\frac{\partial w_{0}}{\partial y} \end{array}\right.$$

The strain-displacement relations of the membrane are:

(3) $$\left\{ \begin{array}{l} \varepsilon_{x}=\varepsilon_{x}^{0}+zk_{x}\\ \varepsilon_{y}=\varepsilon_{y}^{0}+zk_{y}\\ \varepsilon_{xy}=\varepsilon_{xy}^{0}+zk_{xy} \end{array}\right.$$

where $\varepsilon_{x}^{0}$, $\varepsilon_{y}^{0}$, and $\varepsilon_{xy}^{0}$ are the reference surface strains at *z* = 0 defined by:

(4) $$\left\{ \begin{array}{l} \varepsilon_{x}^{0}=\frac{\partial u_{0}}{\partial x}+\frac{1}{2}{\left(\frac{\partial w_{0}}{\partial x}\right)}^{2}\\ \varepsilon_{y}^{0}=\frac{\partial v_{0}}{\partial y}+\frac{1}{2}{\left(\frac{\partial w_{0}}{\partial y}\right)}^{2}\\ \varepsilon_{xy}^{0}=\frac{\partial u_{0}}{\partial y}+\frac{\partial v_{0}}{\partial x}+\frac{\partial w_{0}}{\partial x}\cdot \frac{\partial w_{0}}{\partial y} \end{array}\right.$$

and $k_{x}$, $k_{y}$, and $k_{xy}$ are the membrane curvatures given by:

(5) $$\left\{ \begin{array}{l} k_{x}=-\frac{\partial^{2}w_{0}}{\partial x^{2}}\\ k_{y}=-\frac{\partial^{2}w_{0}}{\partial y^{2}}\\ k_{xy}=-2\frac{\partial^{2}w_{0}}{\partial x\partial y} \end{array}\right.$$

The nonlinear strain-displacement relations given by Equation (4) are those of von Kármán [23]. Due to the constitutive equations, the total stress components for the *i*th layer in Cartesian coordinates are:

(6) $$\left\{ \begin{array}{l} \sigma_{x_{i}}=\frac{E_{i}}{1-\mu_{i}^{2}}\left(\varepsilon_{x}+\mu_{i}\varepsilon_{y}\right)\\ \sigma_{y_{i}}=\frac{E_{i}}{1-\mu_{i}^{2}}\left(\mu_{i}\varepsilon_{x}+\varepsilon_{y}\right)\\ \sigma_{xy_{i}}=\frac{E_{i}}{2\left(1+\mu_{i}\right)}\varepsilon_{xy} \end{array}\right.$$

The strain energy for the *i*th layer is:

(7) $$U_{i}=\frac{1}{2}\underset{V_{i}}{∭}\left(\varepsilon_{x}\sigma_{x_{i}}+\varepsilon_{y}\sigma_{y_{i}}+\varepsilon_{xy}\sigma_{xy_{i}}\right)dV_{i}$$

where *V_i_* is the volume of the *i*th layer. Substituting Equations (3) and (6) into Equation (7) gives:

(8) $$U_{i}=\frac{E_{i}}{2\left(1-\mu_{i}^{2}\right)}\;\iint_{S}\left\{\begin{array}{l} \left(z_{i+1}-z_{i}\right)\left[{\left(\varepsilon_{x}^{0}\right)}^{2}+{\left(\varepsilon_{y}^{0}\right)}^{2}+2\mu_{i}\varepsilon_{x}^{0}\varepsilon_{y}^{0}+\frac{1-\mu_{i}}{2}{\left(\varepsilon_{xy}^{0}\right)}^{2}\right]\\ +\left(z_{i+1}^{2}-z_{i}^{2}\right)\left[\varepsilon_{x}^{0}K_{x}+\varepsilon_{y}^{0}K_{y}+\mu_{i}\left(\varepsilon_{x}^{0}K_{y}+\varepsilon_{y}^{0}K_{x}\right)+\frac{1-\mu_{i}}{2}\varepsilon_{xy}^{0}K_{xy}\right]\\ +\frac{1}{3}\left(z_{i+1}^{3}-z_{i}^{3}\right)\left(K_{x}^{2}+K_{y}^{2}+2\mu_{i}K_{x}K_{y}+\frac{1-\mu_{i}}{2}K_{xy}^{2}\right) \end{array}\right\}dxdy$$

where *S* is the area of the *i*th layer, as shown in Figure 2.

The total potential energy $\Pi_{1}$ of the multilayer membrane as an elastic system is:

(9) $$\left\{ \begin{array}{l} \Pi_{1}=U-W\\ U=\sum_{i=1}^{N}U_{i}\\ W=\iint_{S}pw_{0}\left(x,y\right)dxdy \end{array}\right.$$

where U is the total strain energy, and W is the potential energy of the uniform external load *p* to the *N*-layer membrane.

Since it is extremely difficult to directly solve the nonlinear Equation (9), the Ritz method [24] can be used to approach the analytical approximation under the CMUT’s first order frequency. Both the Galerkin method [25,26] and the Ritz method are effective methods to solve elastic mechanical problems, yet they have different approaches. The Ritz method is a kind of the energy method and based on the principle of least potential energy, while the Galerkin method is a special form of the weighted residual method. For conservative elastic systems whose functional exist, the Ritz method is more efficient and practical. The Ritz method only requires the trail solution to meet the constraint boundary conditions, while the Galerkin methods also requires natural boundary conditions. In our case, the membrane shape is regular, the elastic system is conservative, the functional exist, and the trail solution is available. So we chose the Ritz method over the Galerkin method.

For a clamped square membrane, the boundary conditions are:

(10) $$\begin{array}{ccc} \left\{ \begin{array}{l} u_{0}=v_{0}=w_{0}=\frac{\partial w_{0}}{\partial x}=\frac{\partial w_{0}}{\partial y}=0\\ u_{0}^{t}=v_{0}^{t}=w_{0}^{t}=\frac{\partial w_{0}^{t}}{\partial x}=\frac{\partial w_{0}^{t}}{\partial y}=0 \end{array}\right. & at & \left\{ \begin{array}{l} x=\pm a\\ y=\pm a \end{array}\right. \end{array}$$

Thus, the approximate solutions for the static displacement components can be written as:

(11) $$\left\{ \begin{array}{l} u_{0}=U_{m}h\frac{x}{a}\left(\frac{x^{2}}{a^{2}}-1\right)\left(\frac{y^{2}}{a^{2}}-1\right)\\ v_{0}=V_{m}h\frac{y}{a}\left(\frac{x^{2}}{a^{2}}-1\right)\left(\frac{y^{2}}{a^{2}}-1\right)\\ w_{0}=W_{m}h{\left(\frac{x^{2}}{a^{2}}-1\right)}^{2}{\left(\frac{y^{2}}{a^{2}}-1\right)}^{2} \end{array}\right.$$

where $\frac{2}{3\sqrt{3}}U_{m}$, $\frac{2}{3\sqrt{3}}V_{m}$, and $W_{m}$ are the ratios of the maximum static displacement components in the *x*, *y*, and *z* directions to the total thickness *h*.

Equation (11) is then substituted into Equation (9). According to the Ritz method, the energy expression $\Pi_{1}$ is a function of three coefficients whose numerical values can be determined from the conditions that:

(12) $$\left\{ \begin{array}{l} \frac{\partial \Pi_{1}}{\partial U_{m}}=0\\ \frac{\partial \Pi_{1}}{\partial V_{m}}=0\\ \frac{\partial \Pi_{1}}{\partial W_{m}}=0 \end{array}\right.$$

Equation (12) yields the following set of algebraic equations:

(13) $$\left\{ \begin{array}{l} U_{m}=-\frac{\alpha_{13}W_{m}+\alpha_{14}W_{m}^{2}}{2\alpha_{11}+\alpha_{12}}\\ V_{m}=-\frac{\alpha_{13}W_{m}+\alpha_{14}W_{m}^{2}}{2\alpha_{11}+\alpha_{12}}\\ B_{1}W_{m}+B_{2}W_{m}^{2}+B_{3}W_{m}^{3}=p \end{array}\right.$$

For the cubic equation in $W_{m}$, the coefficients *B*_1_–*B*_3_ are:

(14) $$\left\{ \begin{array}{l} B_{1}=\frac{-2\alpha_{13}^{2}+4\alpha_{11}\alpha_{33}+2\alpha_{12}\alpha_{33}}{1.1378ha^{2}\left(2\alpha_{11}+\alpha_{12}\right)}\\ B_{2}=\frac{-6\alpha_{13}\alpha_{14}}{1.1378ha^{2}\left(2\alpha_{11}+\alpha_{12}\right)}\\ B_{3}=\frac{-4\alpha_{14}^{2}+8\alpha_{11}\alpha_{66}+4\alpha_{12}\alpha_{66}}{1.1378ha^{2}\left(2\alpha_{11}+\alpha_{12}\right)} \end{array}\right.$$

where:

(15) $$\left\{ \begin{array}{l} \alpha_{11}=\sum_{i=1}^{N}\frac{E_{i}h^{2}}{1-\mu_{i}^{2}}\left(z_{i+1}-z_{i}\right)\left[0.85335+0.1016\left(1-\mu_{i}\right)\right]\\ \alpha_{12}=\sum_{i=1}^{N}\frac{0.14225E_{i}h^{2}}{1-\mu_{i}}\left(z_{i+1}-z_{i}\right)\\ \alpha_{13}=\sum_{i=1}^{N}\frac{3.5755E_{i}h^{2}}{a\left(1-\mu_{i}^{2}\right)}\left(z_{i+1}^{2}-z_{i}^{2}\right)\\ \alpha_{33}=\sum_{i=1}^{N}\frac{E_{i}h^{2}}{a^{2}\left(1-\mu_{i}^{2}\right)}\left[0.6986h\left(1+\mu_{i}\right)\left(z_{i+1}^{2}-z_{i}^{2}\right)+8.9165\left(z_{i+1}^{3}-z_{i}^{3}\right)\right]\\ \alpha_{14}=\sum_{i=1}^{N}\frac{0.1201E_{i}h^{3}}{a\left(1-\mu_{i}^{2}\right)}\left(5\mu_{i}-1\right)\left(z_{i+1}-z_{i}\right)\\ \alpha_{66}=\sum_{i=1}^{N}\frac{0.786E_{i}h^{4}}{a^{2}\left(1-\mu_{i}^{2}\right)}\left(z_{i+1}-z_{i}\right) \end{array}\right.$$

where the coefficients $\alpha_{ij}\left(\begin{array}{cc} i=1,\ldots ,6 & j=1,\ldots ,6 \end{array}\right)$ are elements in the compliance matrix of the multilayer membrane. For our case of an isotropic multilayer membrane clamped at its periphery, the compliance matrix can be reduced to a symmetric matrix with six independent elastic constants listed in Equation (15), and other elements are zero [24]. Using the material properties of the CMUT’s layers, Equation (15) can be calculated to determine the static displacements in all three directions.

### 2.2. Response Frequency Analysis

Similarly, the time-dependent vibrating three-dimensional displacements are given by:

(16) $$\left\{ \begin{array}{l} u^{t}\left(x,y,z,t\right)=\left[u_{0}^{t}\left(x,y\right)+\lambda_{x}^{t}z\right]\cos\omega t\\ v^{t}\left(x,y,z,t\right)=\left[v_{0}^{t}\left(x,y\right)+\lambda_{y}^{t}z\right]\cos\omega t\\ w^{t}\left(x,y,z,t\right)=w_{0}^{t}\left(x,y\right)\cos\omega t \end{array}\right.$$

where ω is the angular frequency of the vibrating multilayer membrane. $u_{0}^{t}\left(x,y\right)$, $v_{0}^{t}\left(x,y\right)$, and $w_{0}^{t}\left(x,y\right)$ are the vibrating displacement components in the midsurface in the *x*, *y*, and *z* directions. $\lambda_{x}^{t}$ and $\lambda_{y}^{t}$ are the vibrating rotations of the midsurface about the *x*/*y* axis given by

(17) $$\left\{ \begin{array}{l} \lambda_{x}^{t}=-\frac{\partial w_{0}^{t}}{\partial x}\\ \lambda_{y}^{t}=-\frac{\partial w_{0}^{t}}{\partial y} \end{array}\right.$$

Thus the total displacements in the *x*, *y*, and *z* directions can be written as the summation of the static and the vibrating displacement components:

(18) $$\left\{ \begin{array}{l} u_{T}=\left(u_{0}+u_{0}^{t}\cos\omega t\right)+\left(\lambda_{x}+\lambda_{x}^{t}\cos\omega t\right)z\\ v_{T}=\left(v_{0}+v_{0}^{t}\cos\omega t\right)+\left(\lambda_{y}+\lambda_{y}^{t}\cos\omega t\right)z\\ w_{T}=w_{0}+w_{0}^{t}\cos\omega t \end{array}\right.$$

The strain energy $U_{i}^{T}$ and the kinetic energy $T_{i}$ of the *i*th layer now are:

(19) $$\left\{ \begin{array}{l} U_{i}^{T}=\frac{E_{i}}{2\left(1-\mu_{i}^{2}\right)}\;\iint_{S}\left\{\begin{array}{l} \left(z_{i+1}-z_{i}\right)\left[{\left(\varepsilon_{x}^{0T}\right)}^{2}+{\left(\varepsilon_{y}^{0T}\right)}^{2}+2\mu_{i}\varepsilon_{x}^{0T}\varepsilon_{y}^{0T}+\frac{1-\mu_{i}}{2}{\left(\varepsilon_{xy}^{0T}\right)}^{2}\right]\\ +\left(z_{i+1}^{2}-z_{i}^{2}\right)\left[\varepsilon_{x}^{0T}k_{x}^{T}+\varepsilon_{y}^{0T}k_{y}^{T}+\mu_{i}\left(\varepsilon_{x}^{0T}k_{y}^{T}+\varepsilon_{y}^{0T}k_{x}^{T}\right)+\frac{1-\mu_{i}}{2}\varepsilon_{xy}^{0T}k_{xy}^{T}\right]\\ +\frac{1}{3}\left(z_{i+1}^{3}-z_{i}^{3}\right)\left[{\left(k_{x}^{T}\right)}^{2}+{\left(k_{y}^{T}\right)}^{2}+2\mu_{i}k_{x}^{T}k_{y}^{T}+\frac{1-\mu_{i}}{2}{\left(k_{xy}^{T}\right)}^{2}\right] \end{array}\right\}dxdy\\ T_{i}=\frac{\rho_{i}\omega }{2}\;\iint_{S}\left\{\begin{array}{l} \left(z_{i+1}-z_{i}\right)\left[{\left(u_{0}^{t}\right)}^{2}+{\left(v_{0}^{t}\right)}^{2}+{\left(w_{0}^{t}\right)}^{2}\right]-\left(z_{i+1}^{2}-z_{i}^{2}\right)\left(u_{0}^{t}\lambda_{x}^{t}+v_{0}^{t}\lambda_{y}^{t}\right)\\ +\frac{1}{3}\left(z_{i+1}^{3}-z_{i}^{3}\right)\left[{\left(\lambda_{x}^{t}\right)}^{2}+{\left(\lambda_{y}^{t}\right)}^{2}\right] \end{array}\right\}dxdy\;\sin^{2}\omega t \end{array}\right.$$

where:

(20) $$\left\{ \begin{array}{c} \varepsilon_{x}^{0T}=\frac{\partial }{\partial x}\left(u_{0}+u_{0}^{t}\cos\omega t\right)+\frac{1}{2}{\left[\frac{\partial }{\partial x}\left(w_{0}+w_{0}^{t}\cos\omega t\right)\right]}^{2}\\ \varepsilon_{y}^{0T}=\frac{\partial }{\partial y}\left(v_{0}+v_{0}^{t}\cos\omega t\right)+\frac{1}{2}{\left[\frac{\partial }{\partial y}\left(w_{0}+w_{0}^{t}\cos\omega t\right)\right]}^{2}\\ \varepsilon_{xy}^{0T}=\frac{\partial }{\partial y}\left(u_{0}+u_{0}^{t}\cos\omega t\right)+\frac{\partial }{\partial x}\left(v_{0}+v_{0}^{t}\cos\omega t\right)+\frac{\partial }{\partial x}\left(w_{0}+w_{0}^{t}\cos\omega t\right)\cdot \frac{\partial }{\partial y}\left(w_{0}+w_{0}^{t}\cos\omega t\right) \end{array}\right.$$

(21) $$\left\{ \begin{array}{l} k_{x}^{T}=-\frac{\partial^{2}}{\partial x^{2}}\left(w_{0}+w_{0}^{t}\cos\omega t\right)\\ k_{y}^{T}=-\frac{\partial^{2}}{\partial y^{2}}\left(w_{0}+w_{0}^{t}\cos\omega t\right)\\ k_{xy}^{T}=-2\frac{\partial^{2}}{\partial x\partial y}\left(w_{0}+w_{0}^{t}\cos\omega t\right) \end{array}\right.$$

Then the total potential energy $\Pi_{2}$ of the multilayer membrane is:

(22) $$\left\{ \begin{array}{l} \Pi_{2}=\int_{t_{1}}^{t_{2}}\left(U^{T}-W^{T}-T\right)dt\\ U^{T}=\sum_{i=1}^{N}U_{i}^{T}\\ W^{T}=\iint_{S}p\left(w_{0}+w_{0}^{t}\cos\omega t\right)dxdy\\ T=\sum_{i=1}^{N}T_{i} \end{array}\right.$$

According to Hamilton’s principle [27], Equation (22) should satisfy that for arbitrary *t*_1_ and *t*_2_, $\Pi_{2}$ attains its minimum value. The static deflections $u_{0}$, $v_{0}$, and $w_{0}$ obtained from Equation (9) can be used for the calculations of the vibrating displacements $u_{0}^{t}$, $v_{0}^{t}$, $w_{0}^{t}$ and their angular frequency ω. Again, the Ritz method is utilized to evaluate the desired extreme value of $\Pi_{2}$.

Given the boundary conditions of a clamped square membrane as in Equation (10), the approximate solutions for the dynamic displacement components can be written as:

(23) $$\left\{ \begin{array}{l} u_{0}^{t}=U_{t}h\frac{x}{a}\left(\frac{x^{2}}{a^{2}}-1\right)\left(\frac{y^{2}}{a^{2}}-1\right)\\ v_{0}^{t}=V_{t}h\frac{y}{a}\left(\frac{x^{2}}{a^{2}}-1\right)\left(\frac{y^{2}}{a^{2}}-1\right)\\ w_{0}^{t}=W_{t}h{\left(\frac{x^{2}}{a^{2}}-1\right)}^{2}{\left(\frac{y^{2}}{a^{2}}-1\right)}^{2} \end{array}\right.$$

where $\frac{2}{3\sqrt{3}}U_{t}$, $\frac{2}{3\sqrt{3}}V_{t}$, and $W_{t}$ are the ratios of the maximum vibrating displacement components in the *x*, *y*, and *z* directions to the total thickness *h*.

Together with the Ritz method and Hamilton’s principle, the energy functional $\Pi_{2}$ satisfies the following conditions:

(24) $$\left\{ \begin{array}{l} \frac{\partial \Pi_{2}}{\partial U_{t}}=0\\ \frac{\partial \Pi_{2}}{\partial V_{t}}=0\\ \frac{\partial \Pi_{2}}{\partial W_{t}}=0 \end{array}\right.$$

Equation (24) yields a set of algebraic equations about the frequency, with $U_{t},V_{t},W_{t}$ as the unknown variables. Put these equations into a matrix form, and matrix Equation (25) can be obtained.

(25) $$\left(\left(\begin{array}{ccc} \alpha_{11} & \frac{\alpha_{12}}{2} & \frac{\alpha_{13}}{2}+\frac{\beta_{13}}{2}\\ \frac{\alpha_{12}}{2} & \alpha_{11} & \frac{\alpha_{13}}{2}+\frac{\beta_{13}}{2}\\ \frac{\alpha_{13}}{2}+\frac{\beta_{13}}{2} & \frac{\alpha_{13}}{2}+\frac{\beta_{13}}{2} & \alpha_{33}+\beta_{33} \end{array}\right)-\omega^{2}\left(\begin{array}{ccc} \gamma_{11} & 0 & \frac{\gamma_{13}}{2}\\ 0 & \gamma_{11} & \gamma_{13}\\ \frac{\gamma_{13}}{2} & \gamma_{13} & \gamma_{33} \end{array}\right)\right)\left(\begin{array}{l} U_{t}\\ V_{t}\\ W_{t} \end{array}\right)=0$$

where:

(26) $$\left\{ \begin{array}{l} \beta_{13}=\sum_{i=1}^{N}\frac{E_{i}h^{3}\left(z_{i+1}-z_{i}\right)}{a\left(1-\mu_{i}^{2}\right)}\left(1.2009\mu_{i}-0.2402\right)W_{m}\\ \beta_{33}=\sum_{i=1}^{N}\frac{E_{i}h^{3}\left(z_{i+1}-z_{i}\right)}{a^{2}\left(1-\mu_{i}^{2}\right)}\left[4.7157hW_{m}^{2}-4.1919a\left(z_{i+1}^{2}-z_{i}^{2}\right)W_{m}+a\left(1.2009\mu_{i}-0.2402\right)U_{m}\right] \end{array}\right.$$

(27) $$\left\{ \begin{array}{l} \gamma_{11}=\sum_{i=1}^{N}0.0813\rho_{i}a^{2}h^{2}\left(z_{i+1}-z_{i}\right)\\ \gamma_{13}=\sum_{i=1}^{N}0.2787\rho_{i}ah^{2}\left(z_{i+1}^{2}-z_{i}^{2}\right)\\ \gamma_{33}=\sum_{i=1}^{N}\rho_{i}h^{2}\left[0.3303a^{2}\left(z_{i+1}-z_{i}\right)+0.6605\left(z_{i+1}^{3}-z_{i}^{3}\right)\right] \end{array}\right.$$

where $\beta_{ij}\left(\begin{array}{cc} i=1,2,3 & j=1,2,3 \end{array}\right)$ and $\gamma_{ij}\left(\begin{array}{cc} i=1,2,3 & j=1,2,3 \end{array}\right)$ are the reduced stiffnesses and flexural rigidities in the plane-stress constitutive matrix of the multilayer membrane, respectively.

According to matrix Equation (25), if the operation frequency of the multilayer CMUT membrane is known, the relevant material and geometric parameters can be obtained. Similarly, the frequency of the multilayer membrane can be acquired by using the known material and geometric parameters. In order to acquire the nonzero solution of the matrix Equation (25), the matrix determinant should be zero. During this calculation process, the angular frequency ω of the CMUT membrane can be obtained.

Note that for an isotropic material having elastic symmetry with respect to the midsurface, the values of $\alpha_{13}$ and $\gamma_{13}$ for the static and dynamic displacement calculations are zero, meaning that the coupling rigidities between transverse bending and in-plane stretching will disappear. Thus, it can be predicted that our proposed model will be sensitive to symmetric geometries.

### 2.3. Residual Stress Compensation

According to the plate theory [17], geometries can be divided into a “thin plate” category and a “thin membrane” category, according to their dimension ratios of thickness to minimum lateral dimensions. Between them two, thin membranes suffer more from the residual stress. For the isotropic thin plate without residual stress, the method mentioned above is fully capable to acquire the accurate solutions. For anisotropic thin plates, the stiffness matrix or the compliance matrix needs to be modified [25] (which will not be discussed in this paper). For geometries with residual stress, compensation needs to be added.

As one of the most important mechanical characterizations, residual stress in material is influential and inevitable. During the fabrication process, residual stress has been produced. It can be empirically predicted within a certain range, but the exact value cannot be predicted. On the contrary, it has to be measured by specialized instruments, and the measuring methods include the X-ray, the Raman spectrum, the infrared analysis, and the electron diffraction.

When the CMUT membrane vibrates, the residual stress works as a pre-tension. In order to obtain the accurate predictions of the static deflection and the frequency response, residual stress compensation has to be considered.

CMUT membranes can be regarded as isotropic thin plates/membranes, so the strain in the thickness direction can be ignored ($\varepsilon_{z_{i}}^{0}=0$), and the residual stress can be treated as plane stress. Due to the presence of the residual stress, an additional elastic potential energy can be created during the membrane’s vibration. Let the residual stress for the *i*th layer be $\left(\sigma_{x_{i}}^{0},\sigma_{y_{i}}^{0},\sigma_{xy_{i}}^{0}\right)$, and the vibration displacement be:

(28) V(x,y,z,t)=u(x,y,z,t)i+v(x,y,z,t)j+w(x,y,z,t)k

where u(x,y,z,t),v(x,y,z,t),w(x,y,z,t) are the displacement components in x,y,z directions, and i,j,k are the unit vectors in x,y,z directions.

The strain-displacement relationship of the membrane can be defined by:

(29) $$\left\{ \begin{array}{l} \varepsilon_{x}^{\prime }=\frac{\partial u}{\partial x}+\frac{1}{2}\frac{\partial V}{\partial x}\cdot \frac{\partial V}{\partial x}\\ \varepsilon_{y}^{\prime }=\frac{\partial v}{\partial y}+\frac{1}{2}\frac{\partial V}{\partial y}\cdot \frac{\partial V}{\partial y}\\ \varepsilon_{xy}^{\prime }=\frac{\partial v}{\partial x}+\frac{\partial u}{\partial y}+\frac{\partial V}{\partial x}\cdot \frac{\partial V}{\partial y} \end{array}\right.$$

So the additional elastic potential energy due to the residual stress can be expressed as:

(30) $$U_{ad}=\sum_{i=1}^{N}\frac{1}{2}\underset{V_{i}}{∭}\left(\varepsilon_{x}^{\prime }\sigma_{x_{i}}^{0}+\varepsilon_{y}^{\prime }\sigma_{y_{i}}^{0}+\varepsilon_{xy}^{\prime }\sigma_{xy_{i}}^{0}\right)dV_{i}$$

For actual devices, the residual stress can be measured, and the additional elastic potential energy can be calculated using Equation (30). The energy functional for CMUT’s static and vibrating displacements can be modified as:

(31) $$\left\{ \begin{array}{l} \Pi_{1}^{\prime }=U+U_{ad}-W\\ \Pi_{2}^{\prime }=\int_{t_{1}}^{t_{2}}\left(U^{T}+U_{ad}-W^{T}-T\right)dt \end{array}\right.$$

Through similar calculation processes mentioned above, the static deflection and frequency response of the CMUT membrane with the presence of residual stress will be obtained.

This paper focuses on the static deflection and the frequency response of the CMUT’s multilayer membrane. In the next section, Equation (11) will be used for the central deflection and the deflection profiles under a variety of external pressures. Equation (25) will be used to calculated the frequency of the CMUT’s multilayer membrane by $f=\frac{\omega }{2\pi }$. For actual devices, the residual stress compensation will be done using Equations (28)–(31).

## 3. Validation and Discussion

In order to validate the theoretical analyses obtained above, their results were compared with those from simulations, previous literatures, and experiments. Due to the fact that the square membrane cannot be simplified as 2D axisymmetric models as the circular membranes, 1/4 substructure 3D FEM models were constructed. The cubic mesh was chosen for our case, since it gives higher mesh quality for a reduced computation time.

This section is organized as follows: Firstly, three-layer CMUT membranes are analyzed and modeled by the proposed model, simulations, and previous literatures. The analytical predictions of the static deflection profiles and the frequency responses are compared with simulations for different dimensions under different external pressure values.

Secondly, a double-layer CMUT membrane is designed, fabricated, and tested. The static deflection profiles and the frequency response under the standard ambient pressure are measured, using the Sensofar 3D Optical Profiler (Sensofar, Barcelona, Spain) and the Agilent Precision Impedance Analyzer (Agilent Technologies Inc., Palo Alto, CA, USA). The analytical results predicted by the proposed model with and without the residual stress compensation, the other literatures, and the simulations are compared with the experimental data.

### 3.1. Three-Layer Membrane Case

The three-layer membranes were chosen for their prevalence in the MEMS devices [1,14]. The geometries and dimensions of the three-layer CMUT membranes are listed in Table 1. Due to different fabrication processes, CMUT’s device layer can be either conductive or non-conductive. For CMUTs A and B, the 1st layer (SiN*_x_*) is the insulation layer, the 2nd layer (Al) serves as both the device layer and the electrode, and the 3rd layer (SiN*_x_*) is the passivation layer. For CMUTs C and D, the 1st layer (SiN*_x_*) is the device layer, the 2nd layer (Au) is the top electrode, and the 3rd layer (SiN*_x_*) is the passivation layer.

Note that CMUTs A and B share the same geometry, and their three layers are symmetrical about the midsurface. CMUTs C and D share the other geometry, which is not symmetrical about the midsurface. CMUTs A and C have the same side-length, while B and D have the same side-length. All layers within CMUTs A–D membranes are supposed to be ideally homogenous, isotropic, and without residual stress.

The material properties needed for calculations and simulations are listed in Table 2.

#### 3.1.1. Static Deflection Analysis

**Figure 3:**
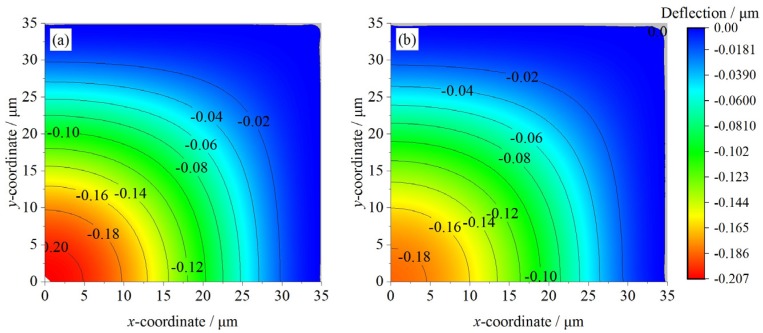
Deflection contours of (**a**) CMUT A for 0.5 MPa and (**b**) CMUT C for 0.5 MPa.

**Figure 4:**
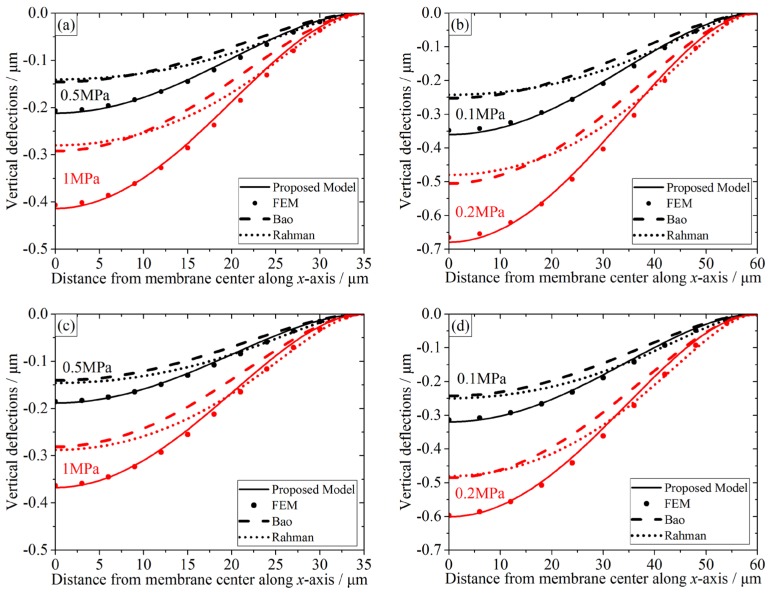
Comparisons of the static deflection profiles of three-layer CMUT membranes predicted by the proposed model, finite element method (FEM), and other literatures, plotted from center to membrane edge. Different colors represent different external pressures. Deflection profiles of (**a**) CMUT A, (**b**) CMUT B, (**c**) CMUT C, and (**d**) CMUT D.

The CMUT’s multilayer membrane can be deflected by the external pressure. The central deflections and the deflection profiles under the external pressure are calculated by our model, Bao [6], Rahman [15], and simulated by FEM. The vertical displacement distribution contours of CMUTs A and C for 0.5 MPa obtained from FEM are shown in Figure 3. The deflection profiles of the three-layer CMUT membranes are provided in Figure 4. The deflection profiles from the membrane center along the *x*-axis are chosen for comparison. Static deflection error analyses of three-layer CMUT membranes for different external pressures are listed in Table 3.

According to plate theory [18], the stiffness of a 70 μm-side-length membrane is bigger than that of a 120 μm-side-length membrane. Thus, for both analytical calculations and FEM simulations, the external pressures are 0.5/1 MPa for CMUTs A and C, and 0.1/0.2 MPa for CMUTs B and D.

The deflection distribution contours in Figure 3 indicate that for the same side-length, CMUTs A and C share a similar deflection tendency, and the largest vertical displacement is exhibited at the membrane center. However, due to the symmetrical geometry, A has a bigger central deflection and smoother deflection variation compared to C. When the external pressure is 0.5 MPa, A’s central deflection is −0.207 μm, and C’s is −0.186 μm. Also, for the same external pressure, a bigger average vertical displacement can be expected for A. For example, the vertical displacements between 15–25 μm for A is −0.15–−0.05 μm, while for C the range is about −0.12–−0.05 μm. Further discussion on this can be obtained by referring to Figure 4 and Table 3.

From Figure 4, it is worth noting that along the *x*-axis away from membrane center, the vertical displacements become smaller until they reach zero at the membrane edges in all cases. The proposed model’s predictions are in strong agreement with the FEM simulations, while the other two show larger deviations. Data from Table 3 indicates that the relative errors of the center deflections between our model and FEM range from 0.71%–3.51%, which is fairly satisfactory. Therefore, it is quite necessary to treat CMUT’s membranes as multilayer models, and our model for CMUT’s multilayer membrane is much more accurate than other analytical models that treat the CMUT membranes as monolayer plates or membranes.

Besides, monolayer models predict smaller deflections. Take CMUT A for 0.5 MPa, for example. Bao’s and Rahman’s models predict similar central deflections, which is around −0.15 μm. However, the FEM simulation is −0.207 μm (Figure 3), and the proposed model’s prediction is −0.212 μm (Table 3). This deviation caused a relative error around 25%, which is not suitable for accurate determination of deflection profiles. Moreover, their predictions about the deflection profiles are similar, too, and Rahman’s profile is smoother than Bao’s due to the electrical coupling coefficients. Similar conclusions could be obtained from other comparisons for the same external pressure in Figure 4 and Table 3.

To make a further discussion on our model’s validation, we focus on the comparisons between the proposed model’s predictions and the FEM simulations.

From Figure 4a and Table 3, it can be observed that for CMUT A, when the external pressure is bigger, the relative error is smaller. At the membrane center (*x* = 0), our proposed model predicts slightly bigger vertical displacement than FEM. For the 0.5 MPa case, the relative error between our prediction and FEM is 2.41%. For the 1 MPa case, the relative error is reduced to 1.82%. Similar conclusions could be obtained from other sub-figures in Figure 4 and Table 3. Thus, the proposed model can maintain accurate predictions for a wide range of external pressures.

Take the deflection comparison between Figure 4a,c for 0.5 MPa, for example. It can be noticed that A has bigger vertical displacement than C, which was proved by Figure 3, too. Also, A’s relative errors are bigger than C. For the 0.5 MPa case, A’s relative error is 2.41%, while C’s is 1.52%. Moreover, for the 1 MPa case, A’s relative error is 1.82%, while C’s is 1.05%. A and C have the same side-length but different geometry, yet their relative errors are similar and small. Thus, the proposed model can obtain stable predictions regardless of the membrane geometry. Comparisons between CMUTs B and D for the same external pressure can lead us to similar conclusions, too.

Comparisons between A and B in Figure 4a,b show that when the side-length is bigger, the relative error is slightly bigger within an acceptable range. For CMUT A, the relative errors are 1.82%–2.41%, while B’s relative errors are 1.96%–3.51%. Due to the dimension error accumulation effect, this relative error deviation between A and B is acceptable. Given the ratios of the membrane thickness to the lateral dimension, both A and B can be categorized as “thin plates” [18]. Thus, the proposed model is suitable for “thin plates” category to obtain accurate deflection profiles. Similar results could be achieved by comparing CMUTs C and D.

**Figure 5:**
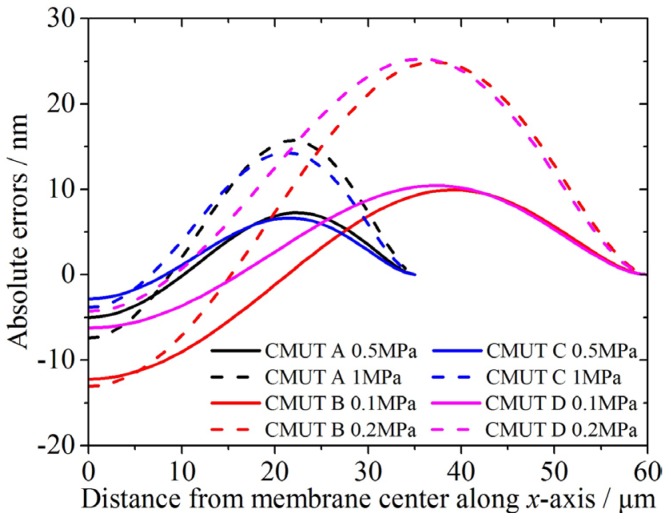
The absolute errors of the static deflection profiles of the three-layer CMUT membranes in relation to FEM simulations.

For a clearer comparison, the absolute errors for the three-layer membranes in relation to the simulation data are shown in Figure 5. Together with Table 3, it can be seen that for symmetrical geometries (A and B), the absolute errors are ranging from 7.261–24.925 nm. For non-symmetrical geometries (C and D), the absolute errors are 6.649–25.265 nm. Besides, for the 70 μm-side-length (A and C) and the 120 μm-side-length three-layer membranes (B and D), the absolute errors are 6.649–15.712 nm, and 9.935–25.265 nm, respectively. These absolute errors are accurate and acceptable for CMUT design and modeling. In Figure 5, there is a peak in every curve. The peak is located around two-thirds of the half side-length away from the membrane center, indicating that the deflection shape function of the membrane can be further modified and improved.

#### 3.1.2. Frequency Response Analysis

**Figure 6:**
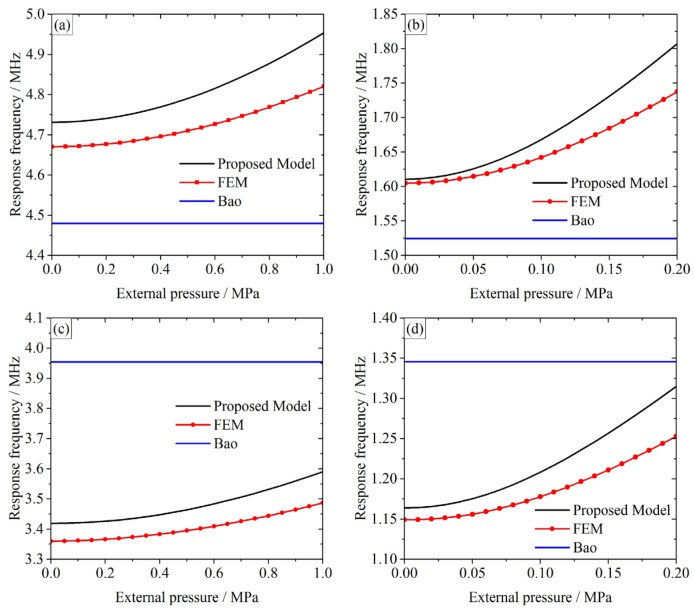
Response frequencies of three-layer CMUT membranes for different external pressures. (**a**) CMUT A, 0–1 MPa; (**b**) CMUT B, 0.1–0.2 MPa; (**c**) CMUT C, 0–1 MPa; (**d**) CMUT D, 0.1–0.2 MPa.

Similarly, a frequency shift can be expected in response to external pressure variation. For the response frequency analysis, Bao’s model [6] is taken as reference. The response frequencies for different pressures are calculated by the proposed model, Bao’s model, and simulated by FEM. The response frequencies of the three-layer CMUT membranes are provided in Figure 6, and the error analyses are listed in Table 4. For the 70 μm-side-length membranes (CMUTs A and C), the external pressure range is 0–1 MPa. For the 120 μm-side-length membranes (CMUTs B and D), the external pressure range is 0.1–0.2 MPa.

From Figure 6, it can be clearly observed that the proposed model’s predictions are in strong agreement with the FEM simulations, while the predictions from Bao’s model [6] show larger deviations. When the external pressures increase, the response frequencies increase, too. However, since the external pressures are not taken into consideration in Bao’s model, the predictions from [6] are invariable, unlike the other two. Therefore, the proposed multilayer model for CMUT membrane is more accurate and suitable for response frequency determination.

Besides, compared to FEM simulations, the response frequencies of the monolayer models could be either smaller or bigger, due to different geometries. Take CMUT A, for instance. The response frequencies using FEM are 4.6703–4.8207 MHz, while Bao’s prediction is 4.4799 MHz, which is smaller than the FEM simulations. On the other hand, for CMUT C featuring A’s side-length but a different geometry, the Bao’s prediction (3.9547 MHz) is higher than FEM simulations (3.3597–3.4868 MHz). Thus, the monolayer models like Bao’s are not accurate enough for response frequency predictions for different external pressures. Similar conclusions could be obtained from comparisons between CMUTs B and D in Figure 6 and Table 4.

According to Table 4, the relative errors between the proposed model and FEM for all cases are ranging from 0.35%–4.96%, which is quite satisfactory. To make a further discussion on our model’s validation, we focus on the comparisons between the proposed model’s predictions and the FEM simulations.

From Figure 6a and Table 4, it can be observed that when the external pressure increases, the response frequency and the relative error increase, too. When the external pressure is 0, the relative error between the proposed model and FEM is the smallest (1.31%). For the 1 MPa case, the relative error is increased to 2.75%, which is still within an acceptable range.

Take the response frequency comparison between Figure 6a,c, for example. A and C have the same side-length but different geometry, and A’s relative errors (1.31%–2.75%) are slightly smaller than C’s (1.77%–2.97%). Similar phenomenon can be observed from comparisons between CMUTs B and D. Thus, the proposed model can accurately predict the response frequencies of different geometries, and more accurate predictions can be expected for the symmetrical geometries.

Comparisons between CMUTs A and B show that when the external pressure is small, B’s relative errors are smaller than A’s; when the external pressure is big, B’s relative errors are bigger than A’s. CMUTs A and B have the same geometry but different side-length, yet their relative errors are within the same acceptable range. Similar conclusions can be obtained by comparing CMUTs C and D. Thus, the proposed model can produce accurate and stable predictions regardless of the dimensions.

### 3.2. Double-Layer Membrane Case

To verify the accuracy of the developed model for actual devices, a CMUT featuring a double-layer membrane has been designed, fabricated, and tested. The two layers are a 1 μm-thick, 70 μm-side-length square polysilicon membrane, and a 0.3 μm-thick, 70 μm-side-length square gold electrode located on top of the polysilicon membrane. The double-layer membrane is separated from the fixed substrate by a 1 μm-deep cavity. Using wafer-bonding technology, the CMUT was fabricated and sealed with a low-vacuum gap (10^−2^–10^−3^ mbar). The Young’s modulus of polysilicon is 160 GPa, the Poisson’s ratio is 0.22. The residual stress is around −200 MPa tested by the Renishaw inVia confocal Raman microscope, so in such plane stress condition, it can be obtained that $\sigma_{x_{i}}^{0}=\sigma_{y_{i}}^{0}=-200\text{ MPa}$ and $\sigma_{xy_{i}}^{0}=0$ in the principal strain direction. These values will be used for the residual stress compensation in Equations (28)–(31). Inside the laboratory, the ambient pressure is around 1.013 × 10^5^ Pa, the temperature is 22 °C, and the granule concentration under 1 μm is less than 1000/m^3^, which are very suitable for delicate experiments. In this subsection, the analytical results from our proposed model with and without the residual stress compensation, the other literatures, and simulations are compared with the experimental data to verify the model’s accuracy.

#### 3.2.1. Static Deflection Analysis

**Figure 7:**
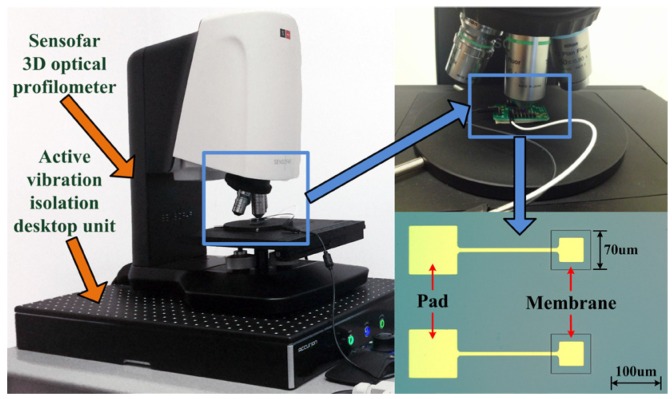
The static deflection testing system.

The static deflection testing system is illustrated in Figure 7. The Sensofar 3D optical profiler (S neox Non-contact 3D Optical Profiler, SensoSCAN 5.3, Sensofar, Barcelona, Spain) is located on the Accurion active vibration isolation desktop unit (Accurion GmbH i4-OD-3173, Accurion GmbH, Goettingen, Germany). An image of the CMUT is obtained using a blue LED light source (the wavelength is 460 nm) for a high lateral resolution.

**Figure 8:**
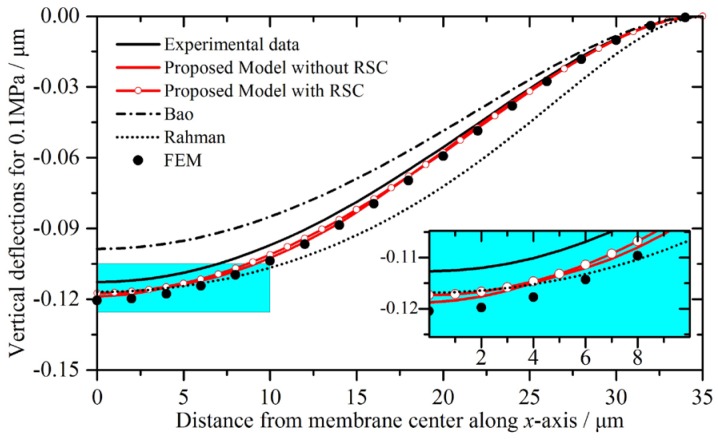
Static deflection profiles and their details of the double-layer CMUT membrane for 0.1 MPa, plotted from center to membrane edge.

The deflection profiles of the double-layer CMUT membrane for 0.1 MPa predicted by the proposed model with and without the residual stress compensation (hereinafter referred as RSC), the other literatures, and the FEM simulations are compared with the experimental data, as shown in Figure 8. The deflection profiles from the membrane center along the *x*-axis are chosen for comparison. The central deflections and error analyses are listed in Table 5.

From Figure 8 and Table 5, it can be observed that the proposed model’s predictions are in good agreement with the FEM simulations and the experimental data. The deviations between the proposed model and the experimental data are reducing along from center to membrane edge. After the residual stress compensation, the central deflection becomes nearer to the experimental data, and the relative errors between the proposed model and the experimental data have reduced from 5.42% to 4.14%. Bao’s predictions are smaller than the experimental data, while the other four results are bigger. Rahman’s deviation at membrane center is the smallest, and the deviations increase along the *x*-axis.

The deviations between the proposed model and the experimental data may be due to the fabrication uncertainties such as manufacturing errors, residual stresses, and heavy doping. They can lower the accuracy of the analytical calculations, since the analytical calculations depend on the geometrical dimensions and material properties. For instance, the heavy doping is necessary for good conductivity, but it also changes the density of the material.

#### 3.2.2. Frequency Response Analysis

The Agilent 4294A Precision Impedance Analyzer (Agilent Technologies Inc.) is used for the frequency response measurements. A 20 V DC voltage was applied and a 0.01 V AC variation was superimposed. The DC bias was chosen based on the following considerations. On one hand, a smaller DC bias voltage can avoid huge electrical coupling effects that can interfere with the validation of the proposed model. On the other hand, a larger DC bias voltage can guarantee accurate measurements and strong electrical signals. According to our calculations, the pull-in voltage of the 70 μm-side-length double-layer membrane is above 200 V, so the DC bias voltage was chosen to be less than 10% of the pull-in voltage.

**Figure 9:**
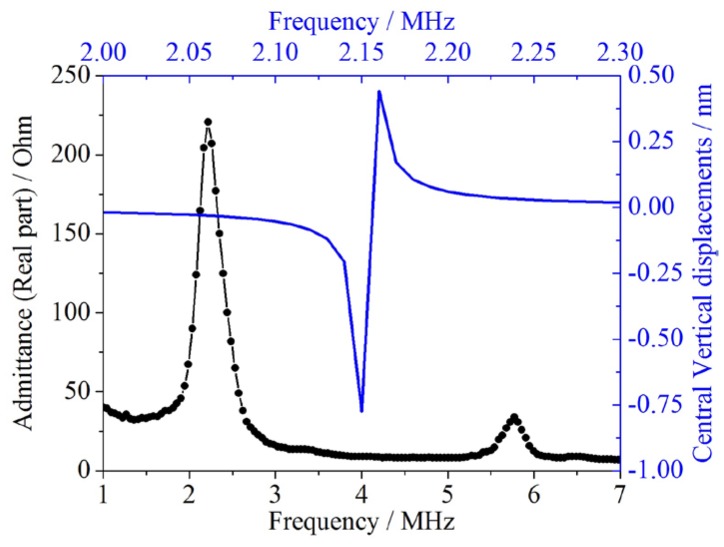
The testing results and FEM simulations of the double-layer CMUT membrane. The black solid line represents the experimental data, arranged against the bottom *x*-axis and left *y*-axis in black. The blue solid line denotes the FEM simulations, arranged against the top *x*-axis and right *y*-axis in blue. The first peak of the experimental data is the first-order response frequency, and the second peak is the second-order response frequency.

The testing results and FEM simulations are illustrated in Figure 9. Since the proposed model directly gives an exact value for the response frequency, the analytical data cannot be illustrated in Figure 9. Instead, the analytical predictions are compared with other values in Table 6.

Note that the experimental data and FEM simulations are arranged using different horizontal and vertical coordinates for a clearer view. This is more suitable, since the impedance measurements represent the real part of the admittance values changing with respect to frequency, while the FEM simulations represent the vertical displacements of membrane center varying with frequency.

Observations from Figure 9 and Table 6 show that for the double-layer CMUT membrane, the proposed model’s predictions strongly agree with the experimental data. With the residual stress compensation, the relative errors between the analytical prediction and the experimental data have reduced from −1.49% to −1.17%, which is quite satisfactory. The FEM simulations provide a similar response frequency and the relative error is −3.02%. Bao’s prediction is bigger than the experimental data, producing a relative error of 6.76%, which also validates the multilayer membrane model. Comparisons and analyses above clearly indicate that the proposed model can offer accurate predictions for the response frequency of the double-layer CMUT membrane, and the residual stress compensation can improve the analytical predictions.

## 4. Conclusions and Further Study

As the crucial vibrating component, the CMUT’s membrane plays an important role in CMUT performance, including the transmitting efficiency and the receiving sensitivity. Accurate determination of the CMUT’s multilayer membrane is vital for design and optimization. Focused as it is on the CMUT’s multilayer characterization, this paper presents a new analytical model for CMUT design and optimization. The theoretical analysis and FEM/experimental verification lead to the following conclusions:

(1) A new analytical model for static deflection and the frequency response of the CMUT’s multilayer membrane under pressure variation has been presented. The derivation of the proposed model and the relevant residual stress compensation method is based on the Ritz method and Hamilton’s principle. The relationships between the external pressure, the static deflection, and the frequency response are obtained.

(2) For three-layer CMUT membranes, the static deflection profile and the frequency response under external pressures have been calculated by the proposed model, other literatures, and FEM simulations. The relative errors are ranging from 0.71%–3.51% for the static deflection profiles, and 0.35%–4.96% for the response frequencies, respectively.

(3) For the double-layer CMUT membrane, the static deflection profile and the frequency response under external pressure variations have been verified by the proposed model, other literatures, FEM simulations, and the experiments. The proposed model can provide accurate predictions. With the residual stress compensation, the relative errors for static deflection have reduced from 5.42% to 4.14%, and the relative errors for frequency response have reduced from −1.49% to −1.17%.

This proposed analytical model can accurately and rapidly predict the static deflection and the frequency response under a wide range of external pressures of the CMUT’s multilayer membrane, and can serve as a reliable reference and an accurate tool for CMUT design and optimization. At the present stage, the model only provides the mechanical characterization of CMUTs, which is basic yet not enough in practice. In our future work, equivalent circuit models will be further developed for the multilayer CMUTs. Besides, CMUTs with rectangular shape membranes will be fabricated and analyzed using our model.

## Figures and Tables

**Table 1:** Geometries and dimensions of three-layer CMUT membranes (μm).

Model Index		A	B	C	D
half side-length		35	60	35	60
material of	1st layer	Silicon nitride (SiN*_x_*)		Silicon nitride (SiN*_x_*)	
	2nd layer	Aluminum (Al)		Gold (Au)	
	3rd layer	Silicon nitride (SiN*_x_*)		Silicon nitride (SiN*_x_*)	
thickness of	1st layer	0.3	0.3	1	1
	2nd layer	1	1	0.3	0.3
	3rd layer	0.3	0.3	0.3	0.3

**Table 2:** Material properties of the three-layer CMUT membranes.

Material	Young’s Modulus (GPa)	Poisson’s Ratio	Density (kg/m^3^)
Silicon Nitride (SiN*_x_*)	250	0.23	3100
Aluminum (Al)	70	0.35	2700
Gold (Au)	70	0.44	19,300

**Table 3:** Static deflection error analyses of three-layer CMUT membranes for different external pressures.

Model Index	Half Side-Length (μm)	Pressure (MPa)	Central Deflection (μm)			Largest Deviation	
			Proposed Model	FEM	Relative Error	Position (μm)	Value (nm)
A	35	0.5	−0.212	−0.207	2.41%	22	7.261
		1	−0.414	−0.406	1.82%	22	15.712
B	60	0.1	−0.360	−0.348	3.51%	39	9.935
		0.2	−0.679	−0.669	1.96%	37	24.925
C	35	0.5	−0.188	−0.185	1.52%	22	6.649
		1	−0.367	−0.363	1.05%	21	14.321
D	60	0.1	−0.320	−0.314	1.98%	37	10.441
		0.2	−0.601	−0.596	0.71%	36	25.265

**Table 4:** Response frequency error analyses of three-layer CMUT membranes for different external pressures.

Model Index	Half Side-Length (μm)	Bao’s Model (MHz)	Smallest Deviation (MHz)			Largest Deviation (MHz)		
			Proposed Model	FEM	Relative Error	Proposed Model	FEM	Relative Error
A	35	4.4799	4.7312	4.6703	1.31%	4.9533	4.8207	2.75%
B	60	1.5244	1.6105	1.6049	0.35%	1.8069	1.7376	3.99%
C	35	3.9547	3.4192	3.3597	1.77%	3.5904	3.4868	2.97%
D	60	1.3457	1.1639	1.1492	1.28%	1.3150	1.2528	4.96%

**Table 5:** Static deflection error analyses of the double-layer CMUT membrane for 0.1 MPa.

Experimental Data (μm)	Methods	Central Deflection (μm)	Absolute Error (nm)	Relative Error
−0.1127	Proposed model without residual stress compensation (RSC)	−0.1188	−6.11	5.42%
	Proposed model with RSC	−0.1174	−4.67	4.14%
	Bao	−0.0986	14.07	−12.48%
	Rahman	−0.1169	−4.17	3.70%
	FEM	−0.1204	−7.76	6.89%

**Table 6:** Response frequency error analyses of the double-layer CMUT membrane for 20 V.

Experimental Data (MHz)	Methods	Frequency Response (MHz)	Relative Error
2.219	Proposed model without RSC	2.186	−1.49%
	Proposed model with RSC	2.193	−1.17%
	Bao	2.369	6.76%
	FEM	2.152	−3.02%
